# Influence of Corrosion on Fatigue of the Fastening Bolts

**DOI:** 10.3390/ma14061485

**Published:** 2021-03-18

**Authors:** Maciej B. Lachowicz, Marzena M. Lachowicz

**Affiliations:** 1 Department of Metal Forming, Welding Technology and Metrology, Faculty of Mechanical Engineering, Wroclaw University of Science and Technology, Lukasiewicza 7-9, 50-371 Wroclaw, Poland; maciej.lachowicz@pwr.edu.pl; 2 Machinefish Materials & Technologies Sp. z o.o., Dunska 13, 54-427 Wroclaw, Poland

**Keywords:** fasteners, high strength bolts, galvanized bolts, corrosion, fatigue failure, thread roots cracking

## Abstract

The aim of the present work was to evaluate high-strength bolt corrosion fatigue based on metallographic examinations. The conducted tests were focused on the analysis of damaged martensitic bolts. It was found that the combined presence of cyclic loads and a corrosive environment was the cause of the accelerated fatigue of the fastening bolts. The tests carried out indicate that the actual operating conditions were different than expected. The corrosion contributed to the loosening of the bolts and initiation of fatigue cracks in the bolt threads. Further damage of the galvanized bolts was caused by fatigue crack growth in their threaded part that propagated towards the centre of the material. Cracks in the zinc coating were transferred to the steel substrate. The corrosion was favored by the oxygen concentration cell and numerous radial cracks appear in the zinc coating. The vibrations accompanying the operation of the wind tower led to their further propagation and the formation of the fatigue fracture in one of the bolts.

## 1. Introduction

Variable and cyclic loads sustained by wind turbine towers make them vulnerable to fatigue [1]. The failure of the mechanical parts of wind turbines has been reported many times [2,3,4,5,6]. Analyzing the causes of their failures is very important to identify potential risk factors affecting the lifecycle of wind turbines. Literature data indicate that 35% of wind turbine failures are due to structural failure or blade failure [2]. Lin et al. [3] were listed as the basic factors affecting the unreliability of this structural elements: technological aspects, low quality of components caused by economic reasons, differences in design standards and climatic location of wind farms. Numerical methods were successfully used to assess possible causes of failure. However, literature data show that the cause of failure is often related to the properties of the materials used for the components [4]. The use of a preload smaller than the recommended one was also noted as a cause of failure [6].

Bolt-on ring flange connections are commonly used to assemble the tubular steel towers that support the wind turbines [1]. They are mounted with high-strength friction-grip bolts. Bolted connections, especially those with tempered high strength bolts, are widely used in engineering constructions and play an important role in their safety and reliability. A statically loaded bolt is held in place by friction forces under its head and on the thread. The biggest problems for the durability of bolted connections are vibrations and cyclic loads, which can gradually reduce the value of the initial stress and lead to self-loosening of the mechanism. In the first 100 cycles, there is more than a 15% reduction in clamp force [7]. Consequently, bolted connections are regarded as unreliable and a regular source of in-service failure. In particular, the resistance to corrosion fatigue of these bolts is responsible for determining the overall reliability and safety of a construction. Therefore, the numerous failures of bolts, and the reasons for these failures, were reported by the authors of papers [8,9,10,11,12,13,14]. Moreover, there has also been research conducted on bolted joints subjected to high cycle transverse loadings [8,15], corrosion [9,10,11,12,13], and surface defects [6,12,14]. The high-strength bolt corrosion fatigue life reduces along with an increase in yield strength, the applied stress or stress amplitude [9]. The accelerated corrosion process causes a reduction of the fatigue strength of joints made of high strength preloaded M12 class 10.9 bolts [10]. The effect of preload on fatigue damage in bolts has also been reported. Excessive corrosion could eventually lead to a reduction in the preload of a bolt as a result of the applied torque [9]. Bolts made of high-strength steel are highly susceptible to stress corrosion cracking and hydrogen-induced cracking [11,13]. For this reason, attempts are made to search for other materials for these applications [13]. The stress distribution in the tension loaded bolt and with nut assembled, favor nucleation of the cracks at the root of the last engaged thread. Technological defects can significantly contribute to this [14]. For this reason, fine particle peening treatment can improve the fatigue strength of the bolt by reducing the surface roughness, increasing the surface hardness, and introducing high compressive residual stresses in the surface [15]. Bolt connections are protected against corrosion by galvanizing. Literature reports indicate that the presence of a zinc coating does not affect the static strength of hot-dip galvanized elements, but there is a reduction in fatigue strength when compared to non-galvanized elements [16,17,18,19,20,21]. Berto et al. [16,17,18,20,21] indicate that this effect is noticeable in the air, but the reduction in fatigue life is very limited compared to uncoated structural elements. In that respect, hot-dip galvanized and uncoated welded joints made by S355 steel are within the acceptable range for welded structural steel [16,18]. Authors also observed a slight difference between the galvanized and non-galvanized samples in their fatigue tests [17]. The situation has been complicated by the presence of a corrosive environment. Elimination of the fatigue limit in air and drastically reduced fatigue life are the two main effects of corrosive environments [19]. Michailidis et al. [22] indicate different mechanisms of fatigue cracking of non-galvanized and galvanized elements. The influence of assembly on the final strength of the tested connections was also found [21]. Berchem [19] proved that the quantity of cracks in the zinc coating increases as a result of applying a preload at very low stress amplitudes and under corrosive conditions. Consequently, it leads to a reduction in the fatigue strength of galvanized steel. The author associates this with a strong localization of the corrosion processes that are taking place. At high stress amplitudes, where the crack initiation takes place quickly, regardless of environmental conditions, the negative influence of the zinc coating is offset by the increase in strength caused by initial stress leading to deformation strengthening of the steel substrate. Initial deformation leads to an increase in the yield strength of the loaded material due to hardening. The same author conducted research in non-corrosive conditions [23]. He showed that the reduction of fatigue strength under these conditions is associated with an easier way to propagate cracks that initiate in the zinc coating and then move towards the steel substrate. Some authors indicate that the reduction in fatigue strength increases with the thickness of the zinc coating [21,24]. The authors of work [24] refer to the limitation of brittle phases in the coating.

The aim of the present work was to evaluate high-strength bolt corrosion fatigue based on metallographic examinations. The conducted tests were focused on the analysis of damaged bolts, which included the assessment of the fracture damage and the testing of the bolt material, while at the same time taking into account the requirements of the EN ISO 898-1 standard.

## 2. Materials and Methods

The research material consisted of two post-operating hexagon bolts that are used in prestressed butt joints in 10.9 HV bolt class according to PN EN ISO 898-1:2013-06, later known as the EN ISO 898-1 standard. The bolts came from a broken flange connection of a wind turbine tower, which was destroyed 1 year after installation. The two twisted flanges of the tower began to buckle during the tilt, leading to the collapse of the wind turbine tower. These bolts were selected from 7 damaged bolts at the stage of macroscopic preliminary examinations. The damage to bolt 1 was the only one that did not lead to a fracture. The remaining fragments of the bolts with heads, which were delivered for testing, were characterized by a similar nature of damage that led to the formation of a fracture. The bolts were tightened with calibrated hydraulic wrenches with a torque from 6 to 6.5 kNm depending on their dimensions. The actual values of the tightening forces applied to the connection were not available to verification. The tower was located in an area with corrosivity class classified as C3 according to PN EN-ISO 12944-2:2018-02. The bolts were not exposed to direct weather conditions, as they were located inside the steel construction of the wind tower.

The following test methods were applied to study the damaged bolts:

1. The measurements of geometry were performed with the 3D GOM Atos Professional V7 SR2 optical scanner (GOM a Zeiss company, Braunschweig, Lower Saxony, Germany), and the obtained models were then compared with the CAD/CAM models of the new bolts with deviations from nominal dimensions according to the the PN EN ISO 898-1:2013-06 standard.
2. The microscopic tests were performed using the Leica M205 C stereoscopic microscope (Leica Microsystems, Wetzlar, Hesse, Germany), Leica DM6000 M metallographic microscope (Leica Microsystems, Wetzlar, Hesse, Germany), as well as the Phenom World ProX scanning electron microscope (Thermo Fisher Scientific, Waltham, MA, USA), equipped with EDX detector on the conventionally prepared metallographic specimens etched with 2% Nital solution.
3. The HV10 hardness measurements were performed using the Vickers method according to the PN-EN ISO 6507-1:2007 standard with the application of a 10 kg load. The test was performed using the Zwick Roell ZHU 8187.5 LKV hardness measuring machine (ZwickRoell, Ulm, Baden-Württemberg, Germany).
4. The HV0.3 micro hardness measurements were performed using the Vickers method according to the PN-EN ISO 6507-1:2007 standard with the application of a 0.3 kg load. The measurements were performed using the Leco LM-248AT micro hardness machine (Leco Corporation, St. Joseph, MI, USA) and the AMH 43 system (Leco Corporation, St. Joseph, MI, USA).
5. The chemical composition was determined by means of the spectral method using the GDS-500A analyser with glow discharge (Leco Corporation, St. Joseph, MI, USA).
6. The static tensile test was performed at room temperature according to the B30 method from the PN-EN ISO 6892-1:2010 standard. The test was performed using the Zwick-Roell Z100 THW (ZwickRoell, Ulm, Baden-Württemberg, Germany) strength testing machine equipped with the automatic extensometer makroXtens^®^ II (ZwickRoell, Ulm, Baden-Württemberg, Germany).
7. The impact resistance test was performed at a temperature of −20 °C according to the PN-EN ISO 148-1:2017-02 standard. The impact energy tests were carried out on non-standard Charpy V type test pieces and performed using the Zwick-Roell RKP 450 181616/2008 impact hammer (ZwickRoell, Ulm, Baden-Württemberg, Germany).
8. The analysis of the deposit, using the method of atomic absorption spectroscopy according to the PN-EN ISO 15586:2005 standard, was performed with the use of the Perkin Elmer model Analyst 600 atomic absorption spectrometer (PerkinElmer, Waltham, MA, USA).

## 3. Research and Discussion

### 3.1. Chemical Analysis

In order to evaluate the chemical composition of the tested bolts’ material, spectral analysis of the chemical composition of the S1 and S2 bolts was performed. The results, as well as the requirements in the standard, are presented in Table 1. It was found that the chemical composition of the tested bolts is typical of steels intended for quenching and tempering, and corresponds to the requirements for bolts of the 10.9 class set by the EN ISO 898-1 standard. The content of Cr, Ni, Mo and V elements may be lower than the specified values if two, three or four of them occur in the steel simultaneously. In this case, when qualifying steel, the limit content of 70% of the sum of the limit content of these individual elements should be additionally taken into account.

### 3.2. Macroscopic Examination

#### Bolt 1

**Figure 1:**
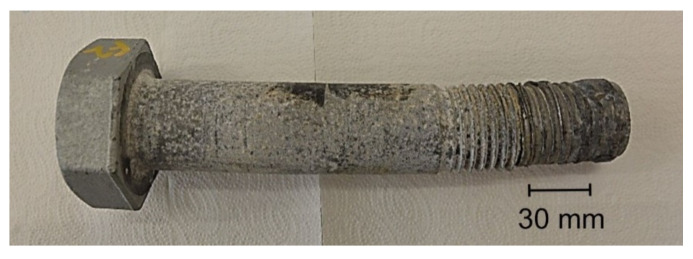
General view of the S1 bolt.

**Figure 2:**
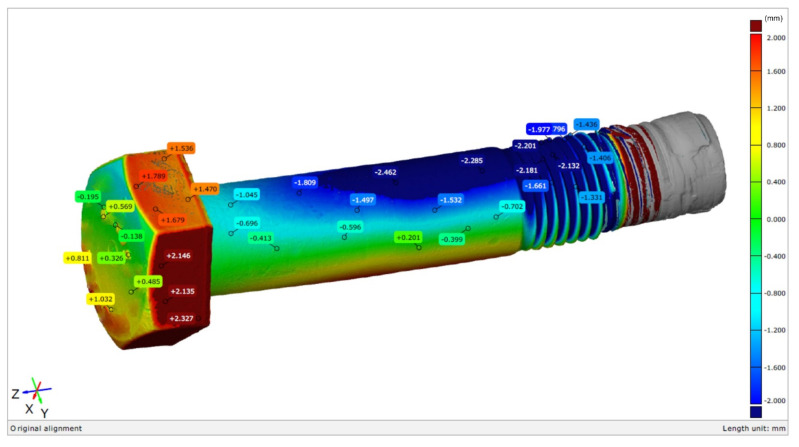
Colored map of deviations for the S1 bolt.

The overall view of the tested S1 bolt is shown in Figure 1. It was characterized with a secondary bend, which resulted in unacceptable shape deviations in the bolt’s shank. The arrow of the bend for the bolt was up to 2.3 mm (Figure 2).

**Figure 3:**
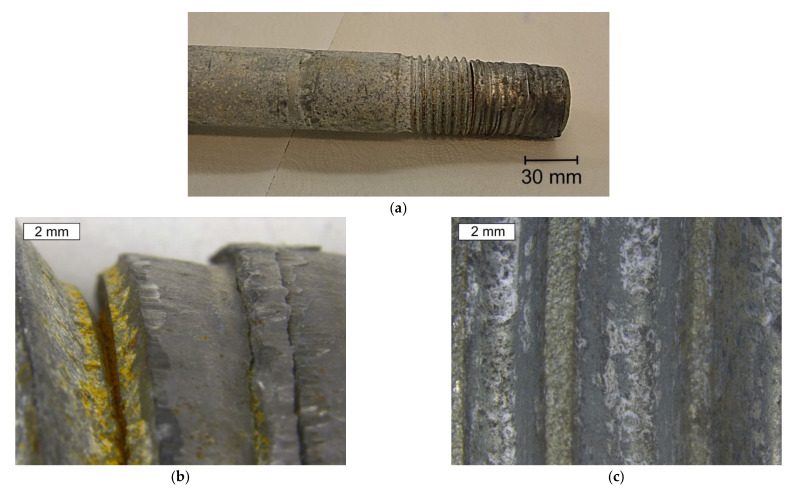
Sheared thread of the S1 bolt: (**a**) General view, (**b**) Cracks in the sheared thread roots, (**c**) Fretting damage of the thread.

**Figure 4:**
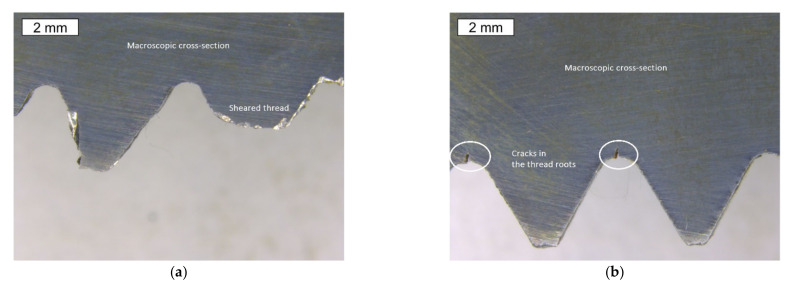
Stereoscopic image of the macro section, which was made at the longitudinal cross-section of the S1 bolt threadinside (**a**) and outside (**b**) of the macroscopic shear area. Visible cracks running along the thread roots.

The bolt was deformed during the catastrophe—the fall of the wind turbine tower. The threaded area had strong deformation as a result of the thread shear (Figure 3 and Figure 4a). The bend of the bolt and thread shearing could only have happened when the two connected flanges of the tower began to split during the tilting and falling of the turbine tower. In such a situation, the nut was not uniformly loaded, which led to a concentration of stress. The pressure is then only present at the small surface of the nut, and a complex state of stresses then begin to act and the tensile and shearing forces, as well as bending moment, appear simultaneously. Such a state of stresses is reproduced in the tensile test of bolts at a wedge, which is described in the ISO 898-1 standard. The trial concerns the shearing head of the bolt, however, the same stress state is evoked when the nut is supported by the wedge. In such cases, thread shear in the bolt or in the nut is very common. In the case of the occurrence of only one-axis tension of a bolt, shearing of the thread should never occur. This is due to the fact that the normalised height of the nut has to provide a much better shear strength of the thread and a stronger surface pressure then the tensile strength of the bolt material. Thread shear could also only occur in the case of the lowering of its strength, e.g., as a result of decarburization. However, this phenomenon in the analysed case did not occur, which was not the case in further tests. Fretting damage of the thread was also observed as a consequence of contact stress occurring in the mating flanks (Figure 3c).

The provided bolt in the area of the sheared thread and in the region of the thread that is normally localized below the nut had numerous deep cracks appearing in the thread roots (Figure 3b and Figure 4b, respectively). The thread roots are the construction notches and they concentrate stresses. This means that this bolt has also suffered fatigue failure, as well as crack initiation in the thread roots. The level of stress and the fatigue crack propagation regions were small enough that they did not lead to fatigue destruction. However, the occurrence of many cracks is a symptom of strong fatigue load, possibly as a result of unsteady bolt vibrations. They can act longitudinally, laterally, or in both directions. Due to alternating horizontal loads, lateral vibrations usually lead to the loosening of the bolts. Longitudinal vibrations pose less risk due to pulsating axial loads. It was reported that high strength bolts are susceptible to high stress concentrations at the root of their thread [14,15,25,26], and especially dangerous tensile stresses occur at the first pair of thread roots [26].

**Figure 5:**
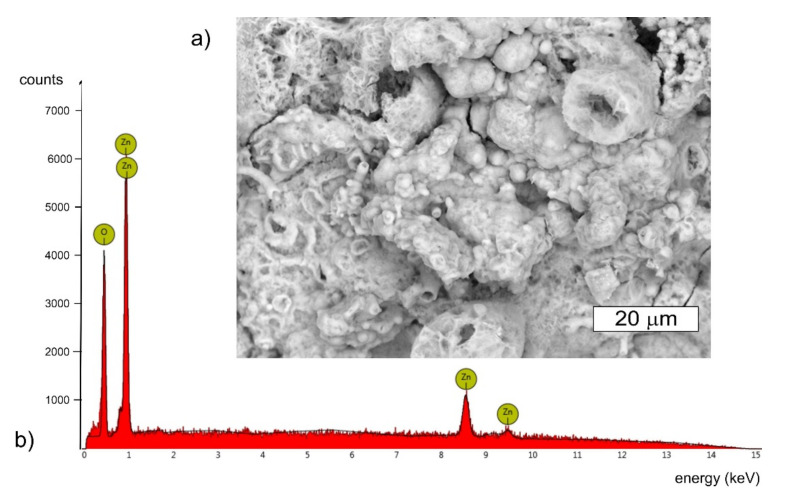
The deposit observed under the S1 bolt head: (**a**) SEM image, BSD (**b**) Characteristic X-ray spectrum for the presented area, SEM/EDX.3.2.2. Bolt 2.

The tested bolt in the location of where the cracks appeared was covered with many corrosion products of white and brown colour (Figure 3b). White rust, also known as white corrosion, is created as a result of corrosion of the zinc coating. The type of formed products is influenced by environmental conditions. The authors agree that after long-term exposure to atmospheric conditions, occurring in the presence of carbon dioxide and in the absence of industrial pollution, the dominant products of the white corrosion of zinc and its alloys are zinc oxide ZnO, zinc hydroxide Zn(OH)_2_, and mainly hydrozincite Zn_5_(OH)_6_(CO_3_)_2_, which is the basic zinc carbonate. When their formation takes place in the presence of chlorine, a simonkolleite with the formula Zn_5_(OH)_8_Cl_2_·H_2_O is also formed [27,28]. The formation of corrosion products may be accompanied by the formation of smithsonite with the formula ZnCO_3,_ or gordaite with the formula Na_4_Zn_4_SO_4_(OH)_6_Cl_2_·H_2_O [27,29,30]. In the EDX spectrum obtained from white corrosion products, no presence of aggressive ions such as chlorides, which could accelerate the corrosion, was observed (Figure 5). However, it is worth mentioning at this point that other bolts from this wind turbine tower were also tested and the presence of chlorine was observed. In the places of cracks, red corrosion of the steel was also observed, which indicates that the zinc layer in these locations was not protecting the steel against corrosion.

The state of the bolt surfaces indicated their relatively strong corrosion, despite the fact that the bolts were exposed to a low corrosive environment and that their service life was short. After a year of operation, the bolts should not show symptoms of coating corrosion, and instead should keep a metallic shine and a tight homogeneous zinc layer. The head of the tested bolt did not show symptoms of white corrosion, but the areas between the bolt’s heads and nuts, which were also in contact with the steel flanges of the tower, showed strong white corrosion. It has to be assumed that electrolytes penetrated between the flanges and the bolt, which stayed in the slots causing strong corrosion of the bolts. The head stayed outside the flange and was self-drying, whereas the bolt fragments remaining in the flanges had restricted air flow. Wetness probably got into the junction of the bolt as a result of flange leakage, but it might also have resulted from temperature changes and moisture condensation inside the turbine tower. The extensive area of white corrosion, however, indicated a prolonged stay of the bolt in a humid environment.

**Figure 6:**
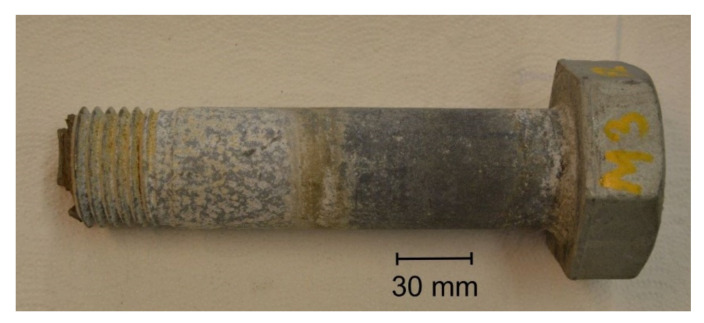
General view of the S2 bolt’s fracture formed in the threaded part.

**Figure 7:**
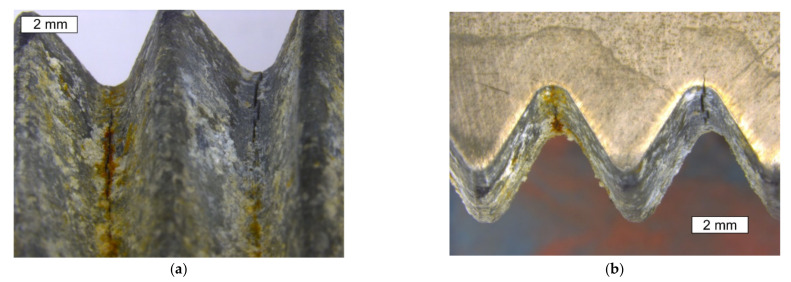
Stereoscopic image of the macro section taken at the longitudinal section of the S2 thread. Visible cracks running along the thread roots, as well as white and red corrosion symptoms: (**a**) area 1, (**b**) area 2.

Macroscopically, the surface showed symptoms of strong white corrosion of the zinc layer of the shank and the threaded part of the bolt (Figure 6). In the location of cracks, red corrosion of the steel was also observed. The head of the bolt was free of corrosion symptoms, as was the case with the S1 bolt. The extensive area of white corrosion indicates the long-term stay of the bolt in wet conditions. It should also be noted that in this case the head of the tested bolt did not show symptoms of white corrosion. It was only the area between the bolt head and the nut, which was in contact with the steel flanges of the tower, that showed strong white corrosion. As a result of observing the S2 bolt, the appearance of cracks was seen in the thread roots located in the area under the nut. In the location of cracks, red corrosion of the steel was observed. The condition of the bolt and cracks observed in the thread roots is shown in Figure 7.

**Figure 8:**
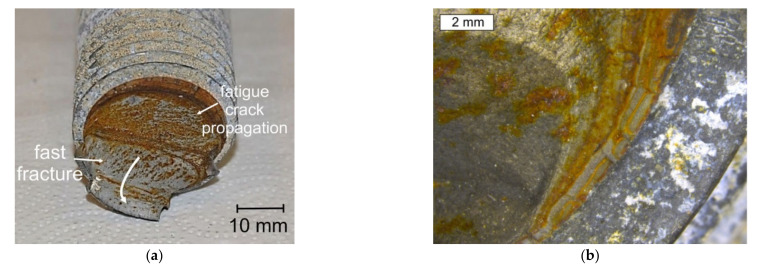
General view (**a**) and stereoscopic image (**b**) of the S6 bolt fracture. The elliptic wrapping of the beach marks.

The failure of the bolts was caused by fatigue crack growth in their threaded part. The general view of the fractures reveals a distinct fatigue crack propagation region and a fast fracture region with pronounced beach marks (Figure 8). The fracture differed from that observed by other authors [11] and caused by hydrogen-induced stress corrosion cracking. The fatigue propagation area was located near the extreme circumference of the bolt. The crack initiated at the thread root and was probably caused by the presence of microcracks, as was also observed in the thread roots of all the tested bolts. Two different rates of fracture development in the fatigue crack propagation region can be observed. In the first phase, this zone did not develop fast and the surface corroded. In the second phase, the rate of crack growth increased, and the fatigue crack propagation region shows much lower features of the corrosion. The bolts showed slight deformation in the fast fracture area, which was also a result of the bolts breaking during the crash (shown by the arrow at Figure 8a).

The failure criterion used in estimating the safety factor of construction materials is the value of the yield stress. In the case of the tested steel, the yield strength is close to the tensile strength. The fatigue fracture surface involves about one half of the bolt’s diameter, which indicates that the safety factor was around 2. The nature of the fatigue crack propagation region and elliptic wrapping of the beach marks indicates that the fracture expanded as a result of tension with mild stress concentrations. The final fracture region shows a brittle–plastic character. The final fracture was most probably created during the collapse of the turbine tower and during the bolt overloading. The bolt had cracks in the fatigue crack propagation region (most probably already before the catastrophe), however, the stresses were not high enough and the cross-section area was still large enough that it did not lead to the bolt’s damage. In the moment of the tower collapse, the stresses abruptly increased, and the bolts were suddenly broken up as a result of overloading, in turn causing the sudden brittle-plastic zones to be created, which are characteristic for heat-treated materials.

### 3.3. Microstructure Observations

#### 3.3.1. Bolt 1

**Figure 9:**
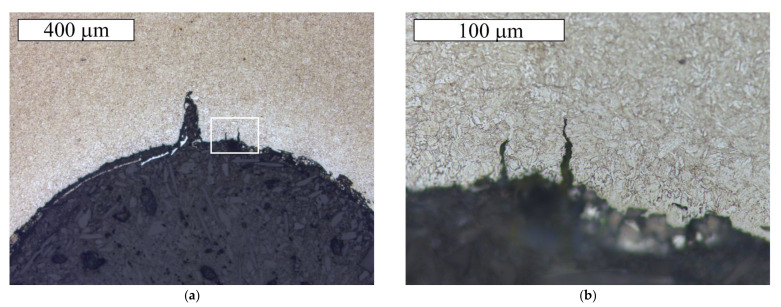
The longitudinal section of the threaded part of the S1 bolt. (**a**) Visible cracks initiated in the thread root, (**b**) The enlarged area indicated by the frame at the Figure 9a Etched state, Light Microscopy.

**Figure 10:**
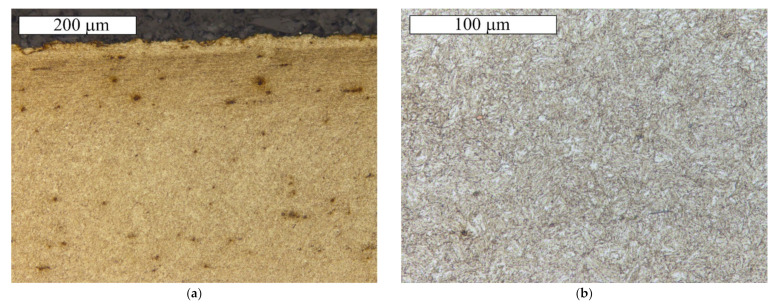
The longitudinal section of the threaded part of the S1 bolt in the surface location: (**a**) Visible martensitic microstructure and a strong plastic deformation of the surface, (**b**) Martensitic microstructure in the core. Etched state, Light Microscopy.

The microscopic observations of the S1 bolt were performed at longitudinal sections of its threaded parts. The observations were conducted in a non-etched and etched state with 2% Nital. Figure 9 presents microscopic images of the cracks observed in the thread roots. In the location of the cracks, no significant decarburisation of the surface was found. Moreover, no irregularities that could possibly influence crack initiation were located. At the base of the microscopic observations, it was found that the maximum allowed value (defined by the ISO 898-1 standard—max. 0.015 mm) of the total decarburization of the tested bolt was not exceeded. The material showed a microstructure of the homogeneous fine-acicular tempered martensite, which is the microstructure typical and proper for quenched and tempered steel parts (Figure 10). Figure 10 presents the microscopic image of the surface section through the threaded part of the S1 bolt in the location where the thread was truncated. The strong plastic deformation of the surface, caused by the action of the shearing force in this location, is visible.

**Figure 11:**
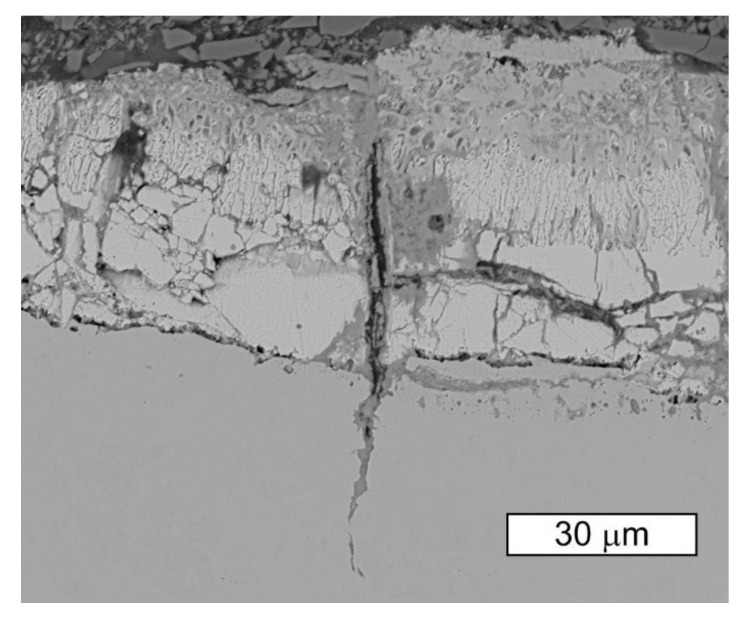
SEM image of the longitudinal section of the S1 bolt thread root (BSD). Visible radial cracks of the zinc coating and steel substrate cracks filled with corrosion products.

The SEM image of the zinc layer observed in the thread root of the S1 bolt is presented in Figure 11. The image shows the crack in the bolt material beginning at the notch surface. It can be observed that the radial crack of the zinc coating also occurs in the place of the bolt material crack. The crack observed in the steel and the crack observed in the coating run almost along one line and act like their own extensions. The opening of the zinc crack suggests that the initiation of the crack happened on the surface of the layer, and also that the resulting crack running through the zinc coating and the screw material is filled with corrosion products. The thickness of the zinc coating at the threads, determined using the microscopy method, was about 40 µm.

The course of the crack initiated in the hot-dip zinc coating is determined by its microstructure. Cracks initiate in the area of the most brittle phases, which are the δ and Γ phases, and then propagate to the ζ phase [31,32,33]. In the case of crack initiation inside the coating, the authors of work [31] did not observe crack propagation to the η phase, which prevents the steel substrate from coming into contact with the corrosive environment. The network of cracks in the area adjacent to the steel substrate may form at the crystallization stage due to the difference in the thermal expansion and elasticity of the steel and the zinc coating [24,34]. The increase in the galvanizing time, leading to the achievement of well-developed hot-dip zinc coatings, promotes the formation of radial cracks [31]. The lead content has also been found to influence the crack rate [35]. This seems to be associated with an increased brittleness of the δ phase in the presence of lead [32]. The number of developing cracks depends on the applied load [24]. The crack development may lead to the exposure of the steel substrate and the lack of cathodic protection of the steel in a corrosive environment. Exposure of the steel surface and zinc corrosion can lead to the absorption of hydrogen.

#### 3.3.2. Bolt 2

**Figure 12:**
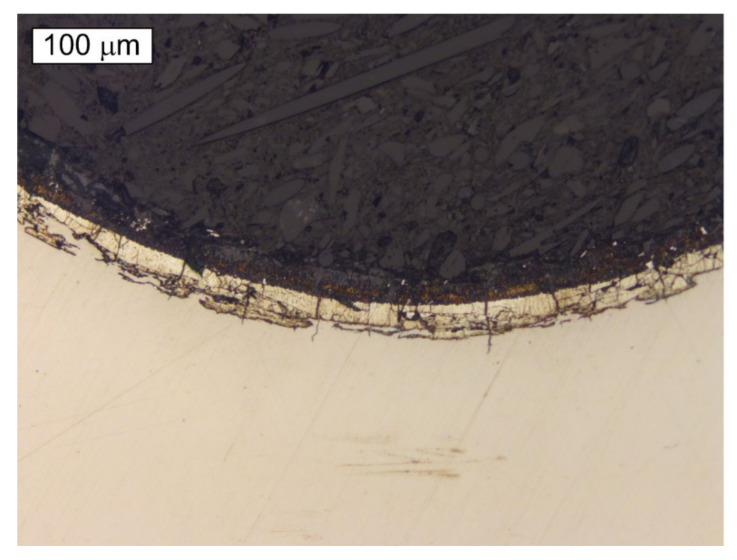
Microscopic image of a longitudinal section of the S2 bolt. Visible cracks initiating in the thread root. Non-etched state, Light Microscopy.

**Figure 13:**
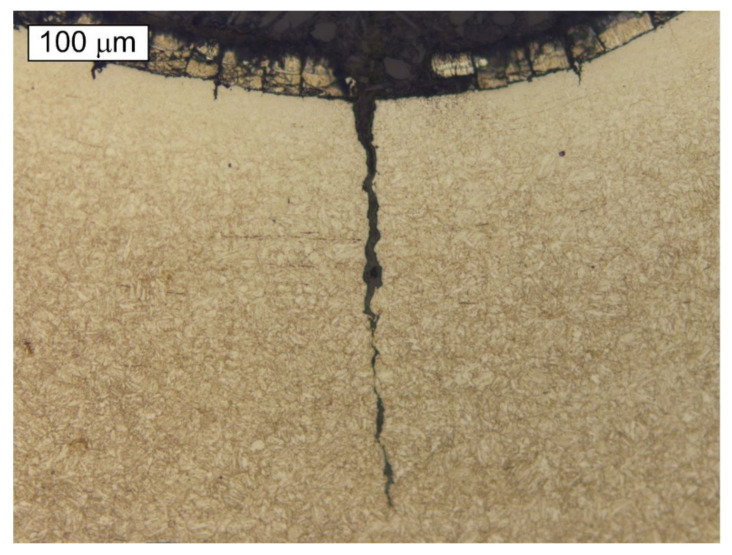
Martensitic microstructure of the longitudinal section of the threaded part of the S2 bolt. Visible cracks initiating in the thread root. Etched state, Light Microscopy.

The microscopic observations of the S2 bolt were performed at the longitudinal sections of its threaded parts in non-etched and etched states. Figure 12 and Figure 13 present exemplary cracks that were observed in the S2 bolt’s thread root. It is very clear that the fracture initiates in the thread root region. Similarly, as in the case of the cracks observed in the material of the remaining bolts, it can be noted that the cracks occurring in the material of the S2 bolt are accompanied by cracks of the zinc coating in the same direction. It was apparent from an examination of the metallographic specimen that crack-like defects were created in the thread roots by a corrosion cracking mechanism. The cracks started at the surface of the root and ran into the bolt’s core.

Similarly, as in the case of the remaining bolts, etching of the S2 bolt material revealed a tempered martensite microstructure, as shown in Figure 13. At the base of the microscopic observations, it was found that the maximum allowed value of the total decarburization of the tested bolt was not exceeded, which is defined by the ISO 898-1 standard (max. 0.015 mm). The requirements concerning the maximum allowable value of decarburization was met by both bolts. Lapping was also observed in the zinc coating, but was not accompanied by cracks.

### 3.4. Mechanical Properties

#### 3.4.1. Tensile Strength

**Figure 14:**
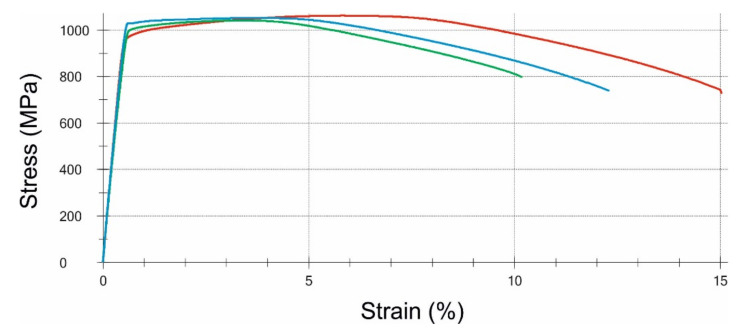
Stress-strain curves obtained for three specimens taken from the S1 bolt.

**Figure 15:**
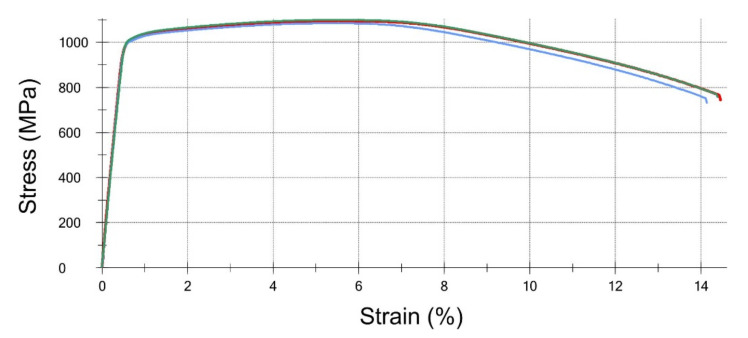
Stress-strain curves obtained for three specimens taken from the S2 bolt.

The samples were taken in accordance with MP1 testing method of the PN-EN ISO 898-1: 2013-06 standard. The tests were performed on flat specimens collected from the bolts in compliance with the EN PN ISO 6892-1:2010 standard. For each of the S1 and S2 bolts, three strength specimens were prepared. Due to the fragment of the S2 bolt being too short, which remained after breaking, it was not possible to perform the tensile test on complete bolts or turned ones in accordance with the ISO 898-1 standard. The reason for this is because the handle part would be too small to keep the bolt in the fixtures of the strength machine, and therefore it would not be possible to determine R_p0.2_ and A. For the S1 bolt, the R_m_ value is oscillating at the limit of the requirements of the ISO 898-1 standard. The difference is rather small, and it has to be considered that the specimens were collected from a damaged bolt, and therefore local weakening of the material cannot be excluded. In terms of strength properties, the S2 bolt met the material requirements for bolts of 10.9 class according to ISO 898-1: 2013-06 (Figure 14 and Figure 15, Table 2).

#### 3.4.2. Hardness Measurements

Hardness measurements using the Vickers method were performed at the transverse section of the threaded part of the bolt in the area between the bolt axis and half of the diameter of the threaded part. The test results are presented in Table 3 with consideration for extended measurement uncertainty. The results were reported as the mean of three measurements. The standard EN ISO 898-1 specifies the range of 320 to 380 HV as the requirement for the hardness of bolts of 10.9 class. The tested material of bolts 1 and 2 had a hardness of 344 ± 4.8 HV10 and 348 ± 6 HV10, respectively. Therefore, both the S1 and S2 bolts met the requirements of this standard for core hardness. High strength steels with a hardness greater than 34 HRC (approx. 340 HV) are susceptible to cracking due to hydrogen embrittlement induced by cathodic protection [13].

**Figure 16:**
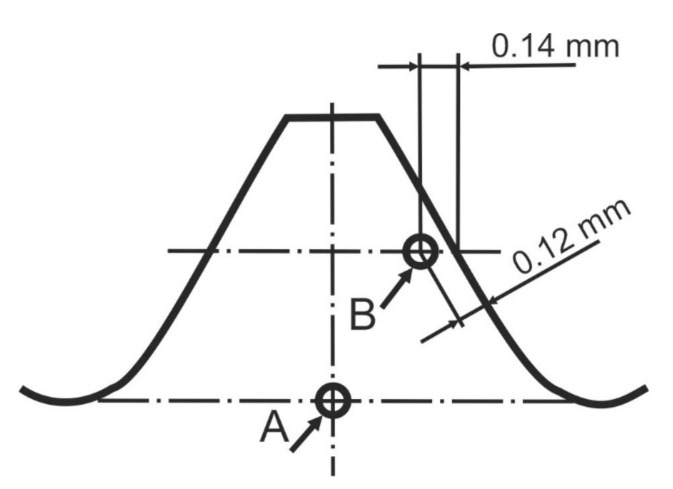
Schematic diagram of the thread section with the marking of microhardness measurement locations in order to state carburization according EN ISO 898-1.

For the bolt of property class 8.8 to 12.9, decarburization and carburization tests should be performed. The measurement of carburization aims to check whether the surface of an element was enriched with carbon during the heat treatment. According to recommendations of the EN ISO 898-1:2013 standard, evaluation of carburization was performed with application of the hardness measurement method at the longitudinal sections of the threaded areas of the S1 and S2 bolts. The assessment is made on the basis of the comparison of the hardness obtained at the height of internal diameter of the thread and the hardness obtained at the height of the thread’s pitch. The standard recommends performing the measurements by means of the Vickers method using a load of 300 g. The HV0.3 microhardness measurements of the longitudinal sections of the S1 and S2 bolts were performed in locations A and B shown in Figure 16. The condition specified by the standard HV(B) ≤ HV(A) + 30 HV is met for both the tested bolts S1 and S2 (Table 2). No carburization of the threads was found.

As an additional condition, the EN ISO 898-1 standard defines the maximum hardness of bolt surfaces. In the case of class 10.9, the hardness of the bolt surface must not exceed 390 HV0.3. The measurement of HV0.3 hardness of the S1 and S2 bolt surfaces was performed on the flat surface of the bolt heads after removal of the zinc coating. The results are presented in Table 3. The surface hardness of the S1 and S2 bolts met the requirements of the standard.

#### 3.4.3. Impact Strength

The Charpy impact strength for the S1 and S2 bolt material was determined by a test performed in compliance with the EN ISO 148-1:2017-02 standard. In accordance with recommendations of the EN ISO 898-1 standard, the impact test was performed at a temperature of −20 °C. For each bolt, the test was performed on two specimens with the Charpy V-notch. As the minimum value of the Charpy Energy (−20 °C) for bolts of 10.9 class, the EN ISO 898-1 standard specifies 27 J. The tested material of the S1 and S2 bolts had an impact strength of 62 ± 1 J and 66 ± 8 J, respectively, and therefore both the S1 and S2 bolt met the requirements of this standard.

### 3.5. Analysis of Deposits on the Surface

**Figure 17:**
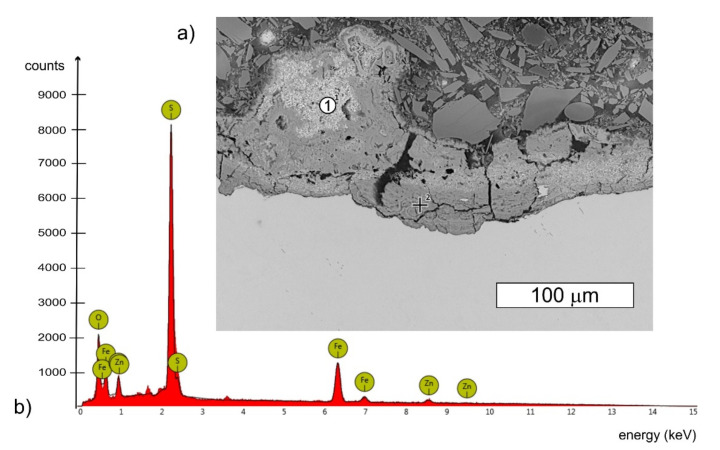
(**a**) SEM image of the deposit observed on the cross-section of the S1 bolt thread surface, BSD (**b**) Characteristic X-ray spectrum obtained for point 1 presented in Figure 17a, SEM/EDX.

**Figure 18:**
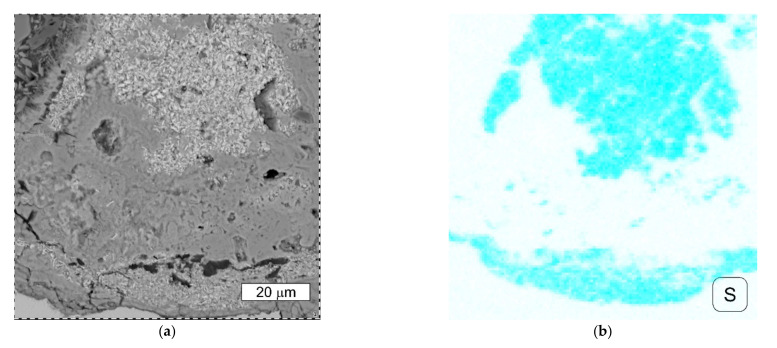
(**a**) SEM image of the deposit observed on the cross-section of the S1 bolt thread surface. An enlarged area from Figure 17a, BSD (**b**) Distributions of sulphur obtained in the mapping performed at the area presented, SEM/EDX.

Torsional and tensile stresses are dominant in the bolts. Each bolt can only be stretched to its yield strength, above which it fails. In order to maximize the possibilities of the bolts, i.e., to achieve maximum tension, and thus the maximum clamping force, without damaging them, it is necessary to minimize torsional stresses in order to maximize tensile stress. This is most effectively achieved by applying lubricant to the bolt thread. An additional benefit resulting from this is the obtaining of repeatability in the obtained values of the clamping force. The bolts should be lubricated with the application of a MoS_2_-based thread lubricant. The deposits present on the bolts provided for the tests were subjected to analysis that aimed to determine the presence of molybdenum in the deposit. On the basis of the performed microanalyses of the chemical composition of the bolt thread surface, the presence of sulphur was found (Figure 17 and Figure 18).

Due to the fact that the overlap in the EDX spectrum between the L series peaks of Mo and the S Kα peak was very pronounced at an applied accelerating voltage of 15 kV, the obtained EDX spectra did not give a clear answer as to whether a lubricant containing molybdenum disulphide MoS_2_ was used. Therefore, the deposits present at the fasteners were subjected to extraction with hexane. After evaporation of the hexane extracts, the dry remnants were analysed using the atomic absorption spectrometry method with electrothermic atomisation—ETAAS, according to the EN ISO 15586 standard. As a result of the performed analysis, the presence of molybdenum was confirmed at the surfaces of all the samples subjected to the tests. This confirms the presence of MoS_2_ molybdenum disulfide, which is a component of grease that lowers friction, on the surface of the fasteners. While the presence of sulfur could be related to surface contamination, the presence of molybdenum is difficult to explain.

## 4. Summary

The following summary of the obtained results can be drawn:

1. The material of the tested bolts met all the quality criteria of the PN EN ISO 898-1:2013-06 standard. All the mechanical properties correspond to the specifications of this standard. Microscopic examinations revealed that the microstructure of the material of both the bolts was typical for tempering martensitic steel. The material properties were not the cause of bolt failure.
2. The bolts, besides fractures and deformations, had deep cracks in their thread roots located in the area below the nut. In the parts of the threads bolted into the nuts, cracks in the thread roots were not observed. Numerous cracks of the bolts appearing in the thread roots outside the nut indicate that the bolts were fatigue cracked. The occurrence of many cracks in each of the bolts is a sign of strong fatigue loading and, possibly, unspecified oscillations of the bolts. The initial cracks then grew subcritically until they reached their critical size. The probable cause of failure could also be a preload less than the recommended one, which could lead to self-loosening of the joint. The corrosion, due to the applied torque, may have reduced the preload in the bolt.
3. During microscopic examinations of the zinc coatings, it was found that numerous radial cracks appear in them (perpendicular to the surface). Their presence in the coatings is not harmful because they do not lower the coating’s adhesion. Moreover, they do not deteriorate the corrosion resistance of the element, because when its surface is wet, zinc becomes a protective anode and iron enters the state of immunity. Steel substrate does not corrode even when moisture penetrates to the substrate. This principle works in the case of most of the radial cracks observed on the bolts. Observations of cracks in the zinc coating appearing at the lateral surfaces of the bolt threads allow for the statement that, in these places, the corrosion of the steel substrate, which is cathodically protected by the zinc coating, did not occur. Similar cracks were observed in the bolt thread roots, however, in these locations the appearance of steel substrate cracks filled with corrosion products was also observed. It cannot be ruled out that hydrogen plays an important role in further crack propagation. However, this is not the only factor. It can therefore be concluded that the internal stresses in the bolts are the additional factor that contributes to the propagation of cracks in the steel substrate. The mechanical factor concerning the development of cracks may be additionally intensified by the fatigue loads. There is also a theory that corrosion products play the role of the wedge-force in the loading process. This contributes to the opening of cracks that then become channels for the transfer of corrosive media.
4. The bolt was subjected to fatigue load, which induced a fatigue crack at the thread’s root that propagated towards the centre and caused the failure. Both the bolt shanks and the threaded parts of the bolts showed symptoms of strong white corrosion of their zinc layer. However, the heads of the bolts and the threaded part protruding from the nut were free of corrosion symptoms. This means that corrosion is favored by the oxygen concentration cell (differential aeration). Under these conditions, the areas where oxygen starvation occurs are anodic, while the areas with free access to oxygen are cathodic.

## 5. Conclusions

The combined presence of stresses and a corrosive environment was the cause of the failure. The bolts were bolted inside the steel construction of the wind tower, which means that they were not exposed to direct weather conditions. The tests carried out indicate that the actual operating conditions were different than expected, as moisture condensed inside the tower, which contributed to corrosion of the bolts. Bolt corrosion was responsible for their loosening and initiation of fatigue cracks in the thread of the bolts. The vibrations accompanying the operation of the wind tower led to their further propagation and the formation of the fatigue fracture in the case of bolt 2.

## Figures and Tables

**Table 1:** Results of the spectral analysis of the chemical composition of the researched bolts.

Symbol of the Element	C	Mn	Si	P	S	Cr	Cu	Mo	Ni	Al	Ti	V	B
Bolt S1	0.32	1.44	0.18	0.010	0.008	0.41	0.09	0.06	0.25	0.03	0.04	<0.01	0.001
Bolt S2	0.33	1.43	0.19	0.010	0.010	0.40	0.09	0.06	0.25	0.03	0.04	<0.01	0.001
Required for class 10.9	0.20–0.55	–	–	max. 0.025	max. 0.025	min. 0.3	–	min. 0.2	min. 0.3	–	–	Min. 0.1%	Max. 0.003

**Table 2:** Summary of the material properties that were obtained from stress-strain curves, and also the requirements according to the EN ISO 898-1 standard.

Material Property	Proof Stress 0.2%, R_p0.2_ (MPa)	Tensile Strength, R_m_ (MPa)	Elongation, A_5.65_ (%)	Reduction of Area, Z (%)
Bolt S1	1003 ± 29	1052 ± 10	12.1 ± 2.4	53.7 ± 3.5
Bolt S2	1012 ± 6	1093 ± 8	13.9 ± 0.2	54.7 ± 2.0
Required for class 10.9	Min. 940	Min. 1040	Min. 9	Min. 48

**Table 3:** Results of the HV0.3 microhardness measurements at the longitudinal sections of the threads and at the head surfaces of the bolts.

Bolt Number	Hardness HV0.3 ± U		
	HV(A)	HV(B)	Surface Hardness
S1	332.3 ± 23.70	324.3 ± 23.70	331.02 ± 26.01
S2	342.5 ± 23.70	324.8 ± 23.70	343.08 ± 24.55

## Data Availability

Data sharing not applicable.
